# Case Report: Use of veterinary cuttable plates to maintain reduction and reinforce polymethylmethacrylate (PMMA) fixation in two canine C2 vertebral fractures

**DOI:** 10.3389/fvets.2025.1534966

**Published:** 2025-04-30

**Authors:** Olivia M. Snead, Andy Y. Law, Megan R. MacRae, Hannah R. Yoder, Karl H. Kraus

**Affiliations:** Department of Veterinary Clinical Sciences, College of Veterinary Medicine, Iowa State University, Ames, IA, United States

**Keywords:** vertebral fracture, cervical fracture, PMMA, case report, reduction, fixation

## Abstract

Veterinary cuttable plates (VCPs) were used to maintain reduction and provide reinforcement in screw and polymethylmethacrylate (PMMA) repairs of C2 vertebral fractures in two dogs. A 6-month-old male intact 3.8 kg Shih Tzu and a 4-year-old male intact 30.5 kg Golden Retriever presented after motor vehicle accidents. Computed tomography (CT) scans revealed fractures of the C2 vertebrae in both dogs. Fractures were reduced and stabilized with a combination of cortical screws, VCPs, and PMMA. The novel technique utilized veterinary cuttable plates positioned between screws cranial and caudal to the fracture for distraction and reduction of the fractures and can be incorporated into the acrylic. Post-operative imaging showed adequate fracture reduction and relief of spinal cord compression. Recheck examinations demonstrated a return to normal and near-normal neurologic function in both patients.

## 1 Introduction

Cervical fractures account for approximately 12%–20% of all vertebral fractures in dogs and cats, with the C2 vertebra most commonly affected (52%–78%) (1–4). In C2 vertebral fractures, the vertebral body or dens is often affected, leading to instability and spinal cord compression (1, 2). The most common cause of vertebral fractures is trauma, such as motor vehicle accidents (41%–63%) (2, 3, 5–7). Clinical signs on presentation range from only cervical pain to non-ambulatory tetraparesis (2–4, 8–10). The relatively large ratio of vertebral canal to spinal cord diameter in this region allows for moderate degrees of fracture fragment displacement with less severe neurological signs compared to other vertebral column segments (2, 7). In contrast, severe displacement of the cervical spinal cord, specifically at the level of C2-3, is often fatal, and these patients do not present for evaluation (2). Though cervical fractures can be seen on radiographs, computed tomography (CT) scans are the imaging modality of choice, as the multiplanar evaluation is sensitive and specific (7, 11, 12). Distraction and reduction techniques for cervical fracture repair include axial traction through digital manipulation or vertebral distractors, small fragment reduction forceps, or the use of Scoville-Haverfield laminectomy retractors (SHLR) (1, 7). Surgical treatment of cervical fractures is warranted if there is instability, spinal cord compression, or deterioration of clinical signs despite conservative management (1, 2, 5–7, 10). Stability is achieved through open reduction internal fixation (ORIF) techniques including ventrally applied screws/pins with polymethyl methacrylate (PMMA), ventrally applied plates with or without PMMA, screws placed in lag fashion, or locking plates (1, 2, 5–7, 10, 13, 14). The goal of vertebral fracture repair is to provide stable reduction and reduced compression without causing further trauma (2).

The purpose of this case report is to describe the use of cortical screws, VCPs, and PMMA to provide an alternative reduction and fixation technique for C2 fractures. Cortical screws were placed in the cranial and caudal fracture fragments with VCPs situated between the screws to maintain reduction and were incorporated into the final PMMA construct for further stability. The technique was used in two canine patients, a 3.8 kg Shih Tzu and a 30.5 kg Golden Retriever, demonstrating the technique’s applicability to a range of patient sizes.

## 2 Case 1 6-month-old male intact Shih Tzu, 3.8 kg

### 2.1 Clinical history

A 6-month-old male intact 3.8 kg Shih Tzu presented to his primary care veterinarian after a motor vehicle accident. The patient exhibited non-weight-bearing left thoracic limb lameness, intermittent right thoracic limb lameness, neck and pelvic pain, and no neurological deficits. The following day, the patient was referred to Lloyd Veterinary Medical Center (LVMC) at Iowa State University for further care. On presentation to LVMC, the patient was laterally recumbent, bradycardic, and hypothermic. The neurological exam showed no cranial nerve deficits, withdrawal reflexes were present in all four limbs, and hyperesthesia. Proprioception, motor, and gait were not evaluated at this time due to the patient’s unstable condition. Left humeral radiographs and CT imaging of the thoracolumbar vertebral column confirmed the presence of a long oblique complete fracture of the left distal humeral diaphysis but did not identify any other abnormalities. Surgical repair of the left humeral fracture was performed, and the patient was hospitalized. Cervical injury was not suspected at this time, as humeral fracture and polytrauma were possible explanations for lateral recumbency.

### 2.2 Physical exam

Following repair of the left humeral fracture, the patient’s neurological status was re-evaluated and showed non-ambulatory tetraparesis, intact withdrawal reflexes in all four limbs, and hyperesthesia. These clinical signs and neurological status prompted further imaging of the cervical vertebral column.

### 2.3 Diagnostic imaging

**Figure 1:**
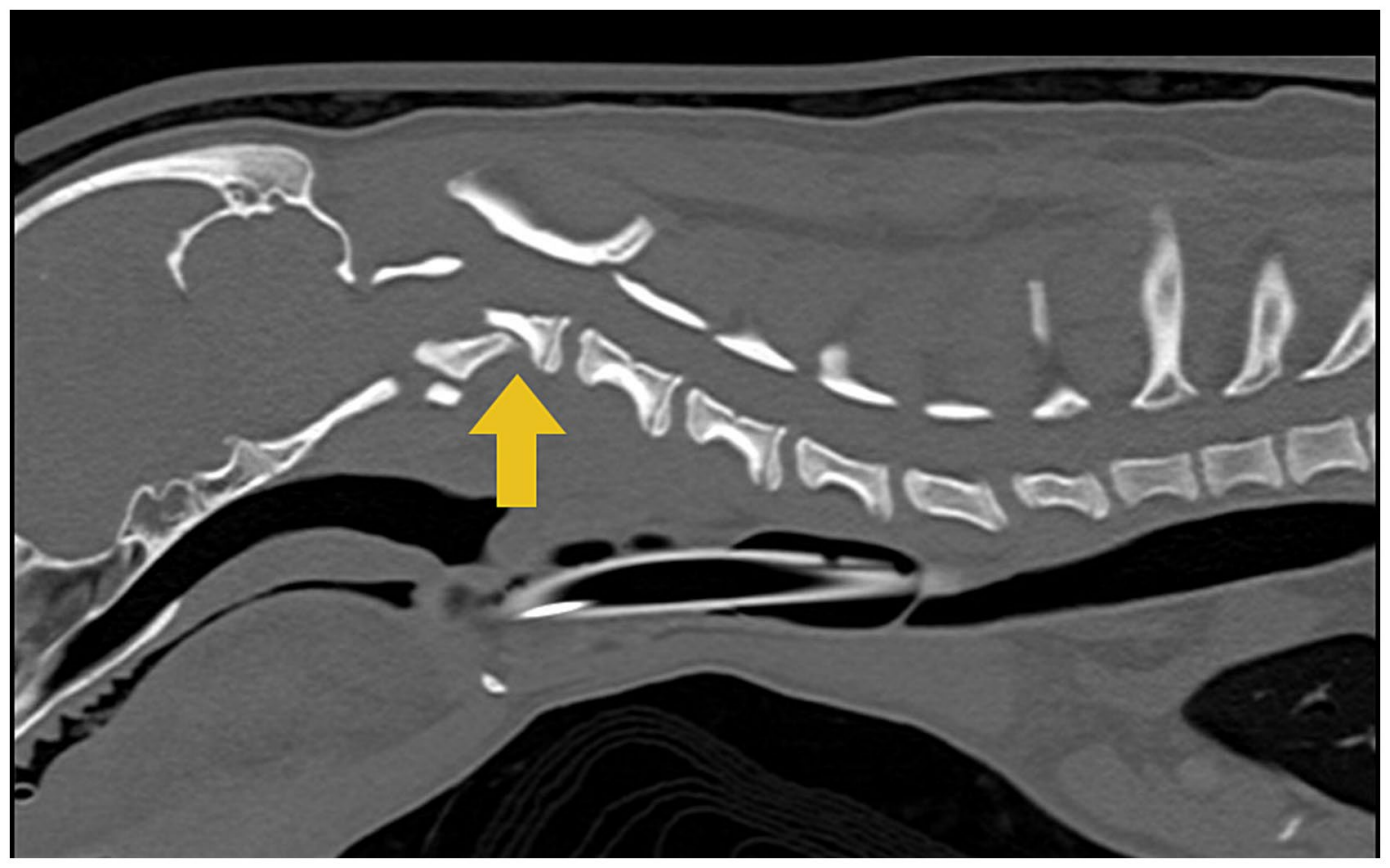
Computed tomographic (CT) image of the cervical region shows a C2 vertebral fracture with the dorsal displacement of the caudal fragment causing spinal cord compression indicated by the yellow arrow.

A CT scan (16-slice Canon Aquilion scanner TSX201A, Canon Medical Systems, Tustin, California) of the cervical vertebral column was performed, which revealed a well-defined and sharply margined oblique fracture of the C2 vertebral body extending in the right caudo ventrolateral to the left cranial dorsolateral oblique direction (Figure 1).

### 2.4 Surgical procedure

**Figure 2:**
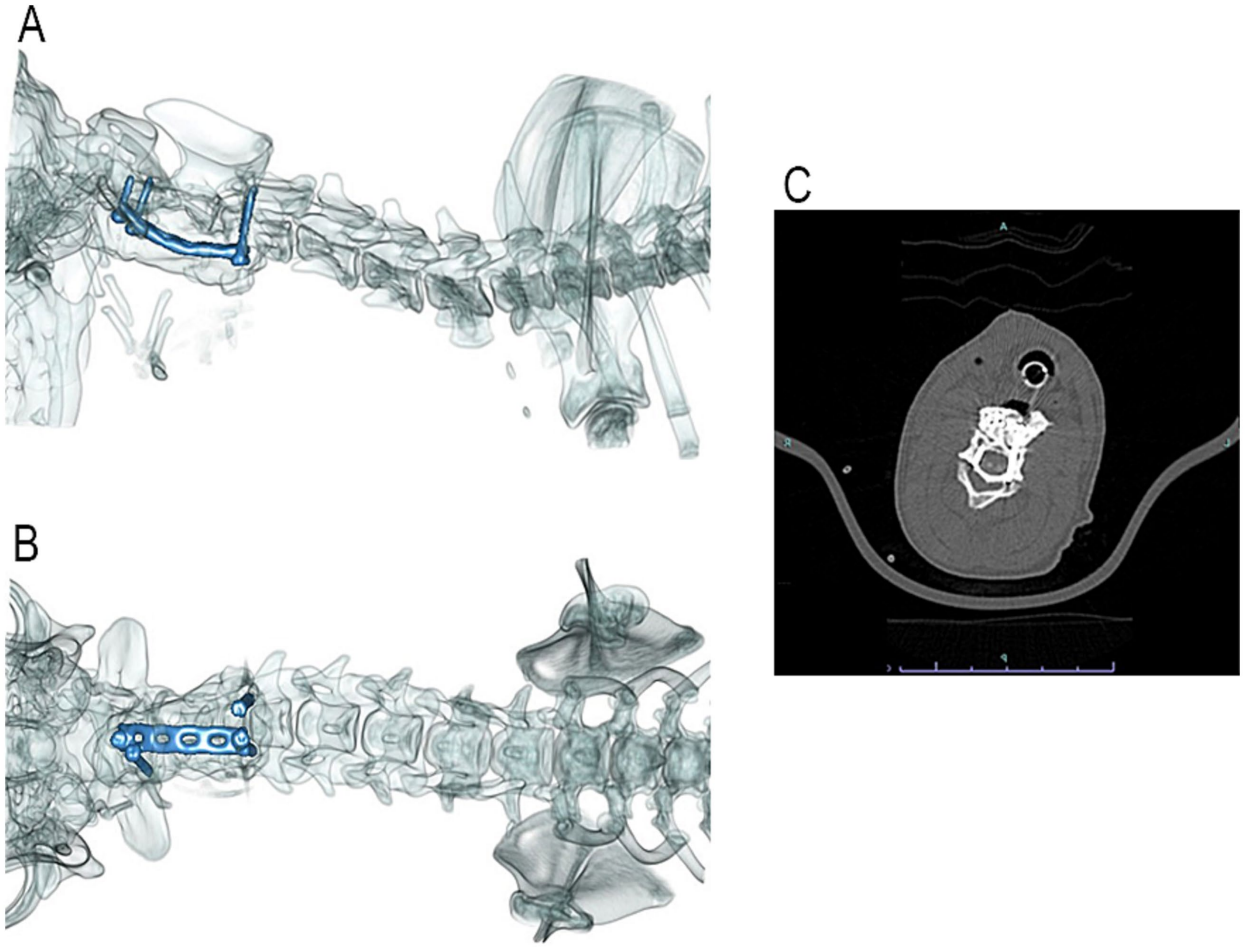
Post-operative 3-D reconstruction of the patient’s cervical vertebral column with lateral **(A)** and ventrodorsal **(B)** views. Two cortical screws are located in the cranial fracture fragment of C2, and two cortical screws are located in the cranial aspect of C3. A VCP was placed diagonally between the cranial and caudal screws and was encased in PMMA. Post-operative transverse CT image **(C)** shows the screws placed the cranial fracture fragment of C2. Though all efforts were made to avoid breaching of the vertebral canal, the right pedicle screw did breach. The left screw penetrated the transverse foramen without adverse consequences in this size patient.

The patient was placed under general anesthesia, prepared for surgery, and positioned to dorsal recumbency. A skin incision was made on the ventral cervical midline with a #10 scalpel blade extending from just cranial to the larynx to the level just cranial to the manubrium. A standard midline ventral approach to the cervical vertebral column was performed, with special care taken to retract and protect the vasculature, recurrent laryngeal nerve, trachea, and esophagus. The C2 vertebral body was exposed through the dissection of the longus colli muscle with a Freer periosteal elevator and bipolar electrocautery forceps. The muscles were retracted with Gelpi retractors, which exposed the fracture margins. The fracture was reduced with a Freer periosteal elevator by gently levering the caudal fracture fragment through the fracture line until fracture reduction was achieved. However, upon reduction, active hemorrhage was noted through the fracture gap, likely from trauma and disruption of the venous sinus. The fracture was thus left mildly under-reduced via gentle traction by an assistant. A 1.1 mm drill bit was used with the corresponding drill guide to place two holes in the cranial fracture fragment with careful attention to avoid the region of the vertebral artery. The drilled holes were measured with a depth gauge, and two 1.5 mm cortical screws were placed in the respective holes, leaving approximately 3 mm of threaded length exposed ventrally. The same procedure was repeated for the screws placed in the cranial aspect of C3. A 2.0 mm 6-hole VCP was cut to create half-circle screw holes on opposite ends of the plate to straddle the screw shafts below the screw heads. The plate was placed between the right cranial screw and the left caudal screw and contoured slightly to the appropriate spacing between the screws to achieve and maintain adequate fracture reduction. The Freer elevator was removed, and reduction of fracture was maintained. PMMA containing cefazolin powder (Fresenius Kabi, United States, Lake Zurich, IL) was used to cover the implants and hold them in place. Closure was routine after copious lavage of the surgical site with sterile saline. Post-operative CT scans revealed that screws were placed ventrally into the vertebral bodies and the pedicles of the cervical vertebrae (Figure 2). Based on the CT images acquired, the positioning of the metal implants was deemed appropriate. There are techniques described to enhance and alter CT images when metal implants are present to determine their exact placement; however, these techniques were not used with these images (14).

### 2.5 Post-operative care

The patient was hospitalized for 5 days prior to transitioning to the in-house rehabilitation facility. Over the following 2 weeks with the rehabilitation service, procedures including massage, passive range of motion, hydrotherapy, and assisted walking, sitting, and standing techniques were performed. The patient progressed from non-ambulatory tetraparetic with weak muscle tone to ambulatory tetraparetic with strong motor tone in all four limbs at the time of discharge.

### 2.6 Recheck exam and long-term follow-up

Recheck examination at 6 weeks post-operatively revealed a neurological status of ambulatory tetraparesis with strong motor movement in all limbs and moderate ataxia. Patellar, withdrawal, and perineal reflexes were intact. Proprioception and overall strength were weaker in the thoracic limbs compared to the pelvic limbs. A CT scan of the cervical vertebral column and radiographs of the left thoracic limb were performed, which showed appropriate healing of both fracture sites with no movement of implants, periosteal reaction, or signs of infection.

Two years postoperatively, the patient was reported to be doing well at home via email and phone communication. The owners reported minimal neurological deficits with mild bilateral forelimb dragging and mild ataxia.

## 3 Case 2 4-year-old male intact Golden Retriever, 30.5 kg

### 3.1 Clinical history

A 4-year-old male intact 30.5 kg Golden Retriever presented to his primary care veterinarian after a motor vehicle accident. At that time, the patient was ambulatory with no other abnormalities noted. The following day, the patient’s neurological status had declined to non-ambulatory tetraparesis, and he was referred to the LVMC for further care.

### 3.2 Physical exam

The patient was laterally recumbent with dull mentation on presentation. Vital signs were within normal limits, and there was no evidence of long bone fractures. Neurologic exam showed intact cranial nerves, withdrawal reflexes present in all four limbs, hyperreflexia of patellar reflexes, and intact deep pain perception. Abdominal and Thoracic Focused Assessment with Sonography for Trauma (AFAST/TFAST) was performed with no abnormalities identified at that time. Advanced imaging was recommended to further assess this patient’s inability to ambulate.

### 3.3 Diagnostic imaging

**Figure 3:**
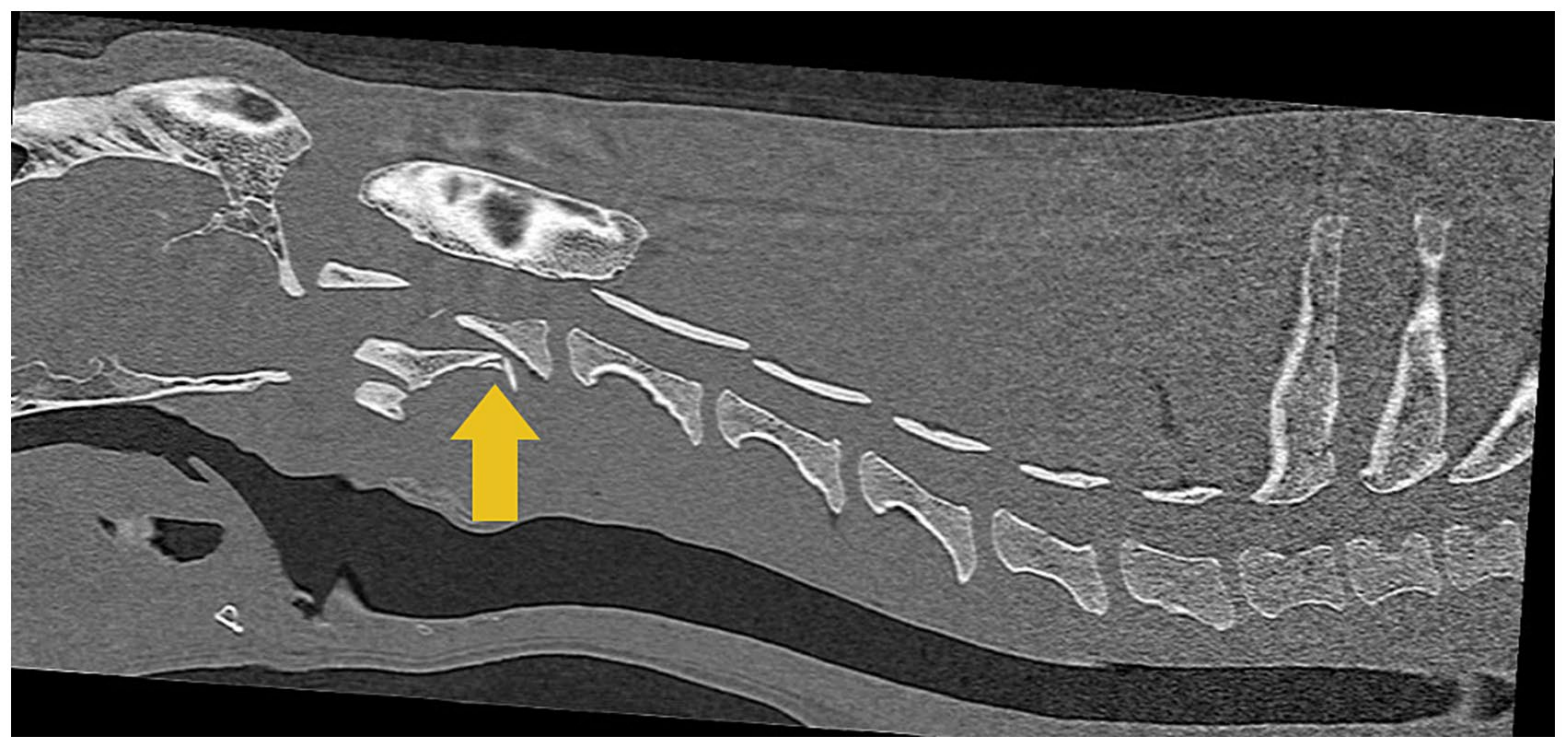
Computed tomographic (CT) image of the cervical region shows a C2 vertebral fracture with the dorsal displacement of the caudal fragment causing spinal cord compression, indicated by the yellow arrow.

A CT scan (16-slice Canon Aquilion scanner TSX201A, Canon Medical Systems, Tustin, California) of the cervical vertebral column was performed, which revealed a well-defined obliquely oriented fracture line that separated the cranioventral portion of the C2 vertebra from the remainder of C2 (Figure 3).

### 3.4 Surgical procedure

**Figure 4:**
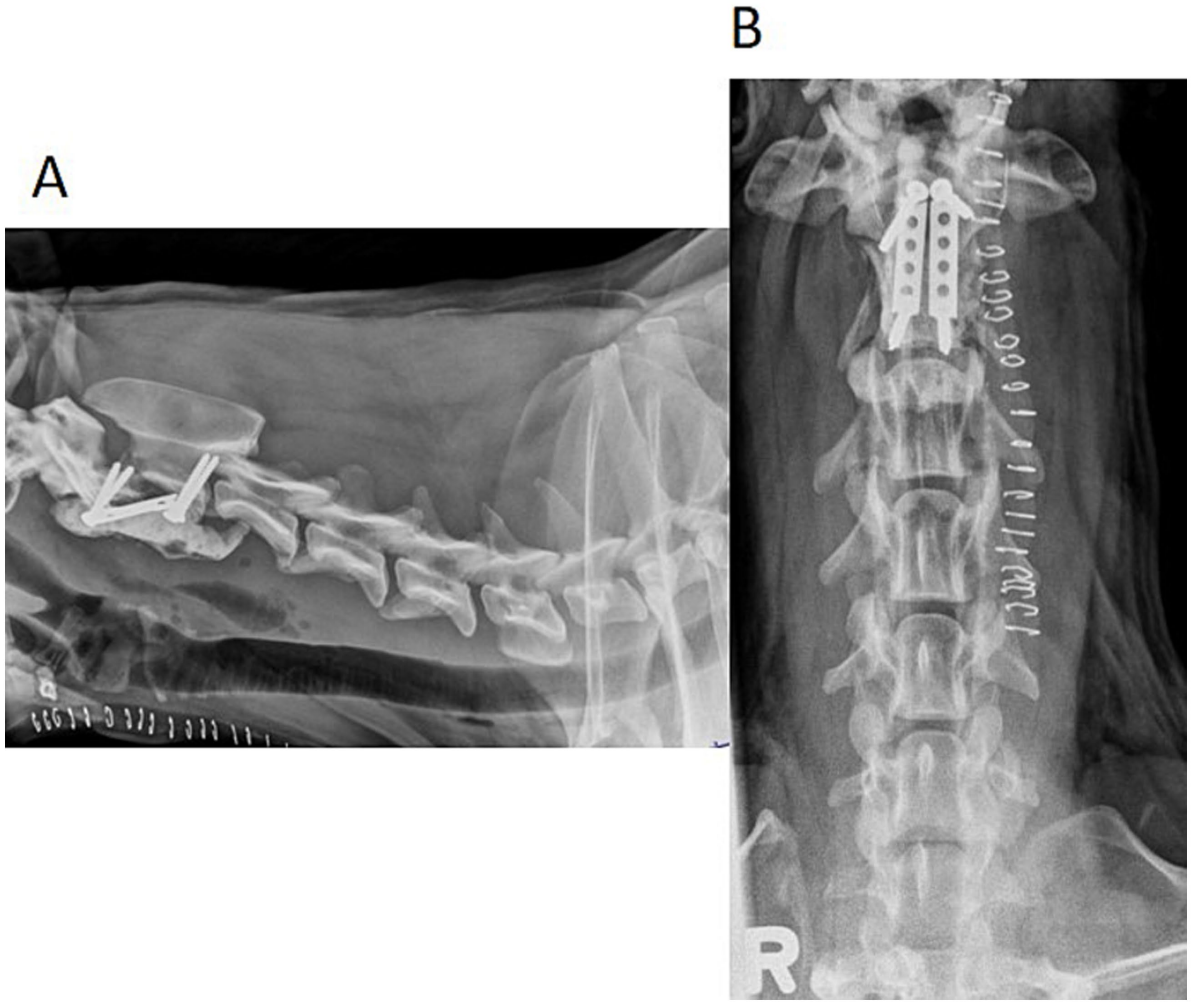
Lateral **(A)** and ventrodorsal **(B)** post-operative radiographs of the patient’s cervical vertebral column. Two cortical screws are located in the cranial fracture fragment of C2, and two cortical screws are located in the caudal fracture fragment of C2. Two VCPs were placed parallel between the cranial and caudal screws and were encased in PMMA.

The patient was placed under general anesthesia, prepared for surgery, and positioned into dorsal recumbency. A skin incision was made on the ventral cervical midline with a #10 scalpel blade extending from just cranial to the larynx to the level just cranial to the manubrium. A standard midline ventral approach to the cervical vertebral column was performed, with special care taken to retract and protect the vasculature, recurrent laryngeal nerve, trachea, and esophagus. The C2–C3 vertebral bodies were exposed through dissection of the longus colli muscle with a Freer periosteal elevator and bipolar electrocautery forceps. The muscles were retracted with Gelpi retractors, which exposed the fracture margins. The fracture was reduced with a Freer periosteal elevator by gently levering the caudal fracture fragment through the fracture line until fracture reduction was achieved. During the reduction, mild hemorrhage was appreciated and controlled with absorbable gelatin (Surgifoam™, Ethicon, Cincinnati, OH) and digital pressure. A 2.5 mm drill bit was used to drill two holes in both the cranial and caudal fracture segments of C2 in a similar fashion as in Case 1. During drilling, mild hemorrhage was encountered and controlled with bone wax (Bone Wax, Ethicon, Cincinnati, OH). The drill holes were measured with a depth gauge, and four, 3.5 mm cortical screws were placed in the respective holes, leaving approximately 4–5 mm of screw exposed ventrally. Two 2.0 VCPs were cut to approximate length between the intended screws in the cranial and caudal fragments with half-circle screw holes on opposite ends of the plate. The plates were placed between the screws in the cranial and caudal fragments with the cut screw holes straddling the screw shafts and contoured to the appropriate spacing between the screws to achieve and maintain adequate fracture reduction. The PMMA was placed over the plate-screw construct and was lavaged with sterile saline to limit thermal damage to the surrounding soft tissues during acrylic hardening. Surgical site closure was routine. Post-operative radiographs revealed that screws were placed ventrally into the vertebral body and the pedicle of the cervical vertebra (Figure 4). In this case, CT would be the preferred imaging modality to determine accurate placement of the screws; however, this was declined by the owner (15).

### 3.5 Post-operative care

The patient was hospitalized for 7 days following fracture repair before transitioning to an in-house rehabilitation facility. Over the following 2 weeks with the rehabilitation service, procedures including massaging, passive range of motion, hydrotherapy, and assisted walking, sitting, and standing techniques were performed. The patient progressed from non-ambulatory tetraparetic with weak muscle tone to ambulatory tetraparetic with strong muscle tone in the hind limbs and moderate muscle tone in the front limbs at the time of discharge.

### 3.6 Recheck exam and long-term follow-up

The patient was lost to immediate post-operative follow-up. Approximately 8 months post-operatively, the owner contacted the hospital and reported that the patient was doing well at home; a neurological status was not specified. Approximately 5 years post-operatively, the patient presented to ISU’s Dermatology service for an unrelated condition. Neurological status was ambulatory with mild ataxia in all four limbs.

## 4 Discussion

### 4.1 Reduction technique

The reduction techniques available for fractures of the cervical vertebral column each have limitations and reported complications (1, 2, 5–7, 10, 13, 14). Axial traction can be applied with either digital manipulation of the maxilla or mandible or by vertebral distractors placed at points cranial and caudal to the fracture site (1). However, these techniques are limited to fractures caudal to the C2 vertebra and, therefore, could not be used with these patients. Small fragment reduction forceps or Freer periosteal elevators can be used to manipulate the fracture fragments to achieve reduction but may be better suited for smaller patients (1). Additionally, once these tools are removed, the fracture may become unstable before the PMMA can provide stability. One reported technique for C2 fracture reduction that does not interfere with the screw/PMMA construct and maintains reduction uses an SHLR retractor, but this has many drawbacks (1). The SHLR requires an incision into the atlantooccipital joint to place the cranial arm of the retractor, which exposes vital anatomy such as the ventral surface of the spinal cord, basilar artery and often results in direct contact with the dura (1). The retractor is sized for human vertebrae, so the use of the retractor requires appropriately sized anatomy and may not be suited for smaller patients (1). This instrument and technique also require a surgeon comfortable with the technical difficulty of the procedure and access to the instrument itself, which limits its use in emergent fractures (1).

The limitations described prompted the authors to explore a novel technique that could be implemented in a variety of patients to maintain reduction without the need for complex instrumentation. VCPs were chosen as they are common, easily accessible, and do not require an incision into vital anatomy to maintain reduction of the fracture during PMMA application and hardening.

Previous techniques utilizing screws as anchor points for orthopedic wire to provide traction to pull the C2 vertebra into the correct alignment have been described (10). This technique requires an assistant to provide constant traction until the Kirschner wires are placed across the C1-2 joint to maintain reduction (10). This technique demonstrates the feasibility of screws as anchor points to provide distraction. In the novel technique described above, screws and VCPs are used similarly to provide a stable, consistent reduction of the fracture once it has been manually reduced. It allows reduction with minimal fracture manipulation and reduces the risk of incorporating unnecessary instruments into the final construct by utilizing the VCPs to maintain reduction.

To the authors’ knowledge, the use of cortical screws placed in the cranial and caudal fracture fragments to serve as anchor points for VCP plates to maintain reduction of cervical vertebral fractures is a technique not previously described. This technique appears to be a viable option for fractures of the C2 vertebrae. While Freer periosteal elevators were used to initially reduce the cervical fractures, the VCPs placed between the screws in the cranial and caudal fragments preserved the reduction so the elevators could be removed without risking loss of reduction. This technique also does not require the use of SHLR, which eliminates the need for an incision into the atlanto-occipital joint capsule and exposure of vital structures and can be implemented in a range of patient sizes.

### 4.2 Fixation and stability of the construct

Common methods of stabilizing cervical fractures include ventrally applied pins or screws with PMMA or ventrally applied plates extrapolated from atlantoaxial instability fixation techniques (1, 2, 6, 7, 10, 13, 16, 17). These techniques can be technically challenging to place in normal patients, and altered anatomy from fractures can increase difficulty and may require specific surgical instruments (1, 2, 6, 7, 10, 13, 16, 17). These instruments may or may not be readily available. The goal of the described surgical technique was to provide a strong and stable fracture fixation using surgical tools and implants that are theoretically readily available to the standard veterinarian. VCPs are readily available and come in a variety of sizes to fit the needs of the patient and their condition.

The use of VCPs in combination with PMMA may improve the rigidity of fixation. In human neurosurgery, techniques have been described that involve the use of PMMA and plates to provide stability for cervical corpectomies for spinal tumors (18). This technique used PMMA for replacement of the vertebral body with the addition of a locking plate and screws placed over the construct (18). The study concluded that the addition of the plate/screw fixation is critical in providing long-term stability of the construct versus PMMA constructs alone (18). This suggests that PMMA constructs with plates are theoretically stronger. However, there are important differences between the two techniques. The plate placement over the PMMA construct in the human construct versus plate incorporation into the PMMA in the novel technique may look similar but could have different biomechanical properties. These two constructs are also used in species with different orientations in space, which alters the biomechanics of the vertebral column.

Biomechanical studies have previously shown that metal implants such as screws plus PMMA fixation techniques bear higher maximal loads, they can withstand higher flexural loads, and provide greater rigidity during constant and gradually increasing loads in cervical constructs (19, 20). It could be extrapolated that constructs with plates, screws, and PMMA could be mechanically stronger with the addition of the VCPs to the construct, but further biomechanical studies are needed. Another study used a similar construct in the C4 to C5 cervical vertebrae to stabilize the vertebral motion of this section (21). This study showed that the screw-bar-PMMA construct increased the PMMA stiffness, which allowed for higher bending moments before implant failure when compared to the traditional pin-PMMA constructs (21). While the construct style used in these two patients is not identical to the construct styles described in the studies, the similarities can be extrapolated, and the biomechanical properties can be applied to the novel construct. It is also important to note that in Case 1, the caudal screws were placed in the cranial aspect of C3, but not in the caudal fracture fragment of C2. This was chosen as the caudal fracture fragment was too small to accommodate the implants. This could potentially put excess strain on the implant and affect the long-term outcome of the construct.

## 5 Conclusion

The two cases presented demonstrate an alternative technique for the reduction and fixation of C2 vertebral fractures not previously reported. The use of VCPs served as a method of maintaining reduction and may contribute positively to the PMMA and screw-plate constructs. The favorable improvement following surgical stabilization, as reported and found throughout long-term follow-up, supports the applicability of this technique in the repair of C2 vertebral fractures in both large and small breed dogs.

Limitations of this report include the limited number of cases presented and its retrospective nature. Further studies would be required to evaluate the technique in a larger subset of patients and the biomechanical properties of the reported construct.

## Data Availability

The original contributions presented in the study are included in the article/supplementary material, further inquiries can be directed to the corresponding author.
